# Mandibular Metastasis of Silent Papillary Thyroid Carcinoma: A Rare Case Report with Review of the Literature

**DOI:** 10.1155/2020/8683465

**Published:** 2020-04-05

**Authors:** Shahzad Gholami, Mahin Bakhshi, Saede Atarbashi-Moghadam, Hassan Mir Mohammad Sadeghi, Arezoo Rahimzamani

**Affiliations:** ^1^Department of Oral Medicine, School of Dentistry, Shahid Beheshti University of Medical Sciences, Tehran, Iran; ^2^Department of Oral and Maxillofacial Pathology, School of Dentistry, Shahid Beheshti University of Medical Sciences, Tehran, Iran; ^3^Department of Oral and Maxillofacial Surgery, School of Dentistry, Shahid Beheshti University of Medical Sciences, Tehran, Iran

## Abstract

Metastasis to the jaws is a rare event; however, it has great importance because it may be the only symptom of an undiagnosed underlying malignancy. Papillary thyroid carcinoma, the commonest histopathologic variant of thyroid cancer, has minimal potential for distant metastasis, and most reported metastatic thyroid carcinomas of the oral cavity have been follicular thyroid carcinoma. The aim of this article is to present a rare case of metastatic papillary thyroid carcinoma, which presented itself as a painless expansion in the anterior region of the mandible in a 56-year-old female, and to review previously published cases of this type of cancer. Reporting rare cases such as this one, together with reviewing previous reports of related rare diseases, will expand the body of knowledge about these uncommon lesions.

## 1. Introduction

Metastases to the oral cavity comprise about 1% of all the oral malignancies and have been mostly detected in the jaws [1]. Metastasis to the jaw has a marked predilection for the posterior region of the mandible, which is rich in red bone marrow and sinusoidal vascular spaces that may be the cause of tumor deposition [1, 2]. Jaw metastasis is more common among older adults (mean age = 53.4 years) and has a male predilection [1]. Nearly 23% of oral metastases are the first sign of an occult malignant tumor elsewhere in the body [2, 3]. The migration can occur via the bloodstream or the lymphatic pathways. The origin of oral metastasis could be different based on gender [2]. Cancers of the lungs, the liver, and the prostate are the source of most jaw metastases in men, and their origin in women are the breasts, the thyroid gland, and the lungs [1]. Thyroid cancers have accounted for up to 6% of the oral cavity metastases [2, 4], occurring more frequently in follicular variants [5]. Oral metastasis is frequently associated with poor prognosis and quality of life [2], and it usually develops at the late stages of the underlying disease [1]. Thus, oral health care professionals have a crucial role in the diagnosis and management of cancer patients, particularly those with silent primary malignancies [1]. The purpose of the present report is to describe clinical, radiographic, and histopathologic features of mandibular metastasis of a silent papillary thyroid carcinoma in a 56-year-old female. It was also considered pertinent to review previously published reported cases.

## 2. Case Report

**Figure 1:**
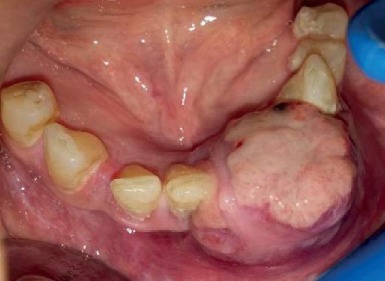
An ulcerated mass in the anterior region of the mandible.

A 56-year-old female was referred to the Department of Oral Medicine, Shahid Beheshti University of Medical Sciences (Tehran, Iran) for evaluation of a rapidly growing painful expansion in the left side of her mandible which by her report had been present for 20 days. The patient reported traumatic luxation of her lower left central and lateral incisors due to a fall and their subsequent extractions by a general dentist. The extraction site had not healed properly and had been expanding. The lesion had been interfering with her eating. Intraoral examination revealed a diffuse swelling in her left anterior mandible measuring 3 × 3 cm and extending from the right central incisor to the left canine. The overlying mucosa was erythematous, ulcerated, and necrotic. The lesion was soft to firm in consistency on palpation (Figure 1).

The patient also suffered from hypertension, diabetes mellitus, and bowel disease.

**Figure 2:**
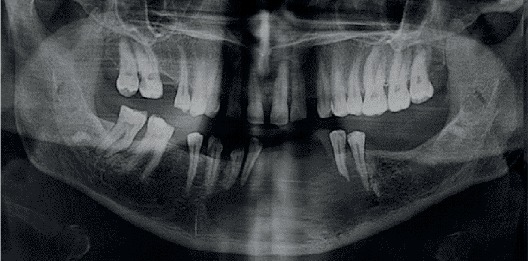
The panoramic radiograph shows an ill-defined unilocular radiolucency extending from the right lateral incisor to the left second premolar, as well as root resorption of teeth 20, 21, and 22.

**Figure 3:**
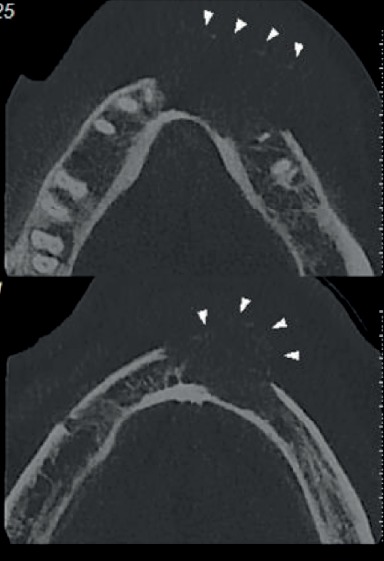
CBCT revealed a large destructive lesion with irregular borders associated with a soft tissue mass causing extensive perforation of the buccal cortical plate.

The panoramic radiograph showed a unilocular radiolucency with ill-defined borders from the right lateral incisor to the left second premolar. Cone beam computed tomography (CBCT) revealed a large destructive lesion with irregular borders associated with a soft tissue mass extending from tooth #26 to the distal of tooth #20, with extensive perforation of the buccal cortical plate. Thinning and erosion of the lingual cortical plate and root resorption of teeth #22, #21, and #20 were also seen (Figures 2 and 3).

**Figure 4:**
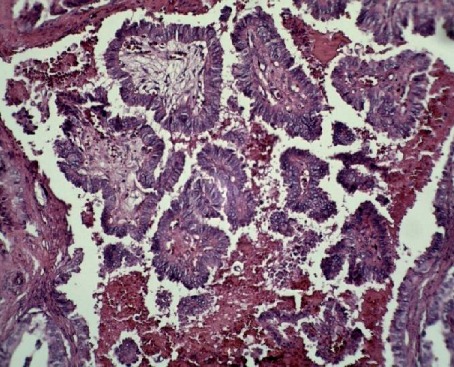
Microscopic sections showed papillary projections with fibrovascular cores lined by cuboidal and columnar cells (H & E ×100).

**Figure 5:**
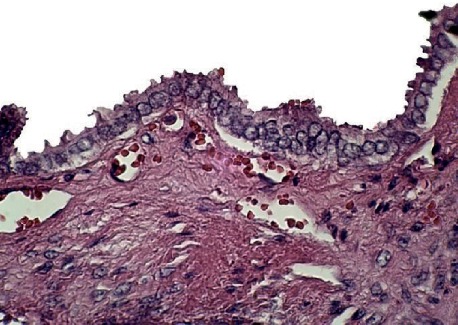
The nuclei showed ground glass appearance (H & E ×400).

There was no cervical lymphadenopathy. According to both clinical and radiographic features, differential diagnoses of aggressive central giant cell granuloma, lymphoma, odontogenic carcinoma, and metastatic neoplasms were considered. The mandibular mass was biopsied, and microscopic sections showed a malignant epithelial neoplasm composed of nests, tubules, and cystic and papillary structures with fibrovascular cores lined by cuboidal and columnar cells. The nuclei showed a ground glass appearance (Figures 4 and 5). Some eosinophilic colloid-like material, hemorrhage, and inflammatory cell infiltration were also seen. The tumor was covered by ulcerated epithelium.

Based on microscopic findings, overall diagnosis of “papillary carcinoma” was rendered. Follow-up immunohistochemical (IHC) staining for TTF1, mammaglobin, CK7, and CK20 was requested for further evaluation and makes difference between metastatic tumors such as papillary thyroid carcinoma (PTC) and papillary breast cancer.

**Figure 6:**
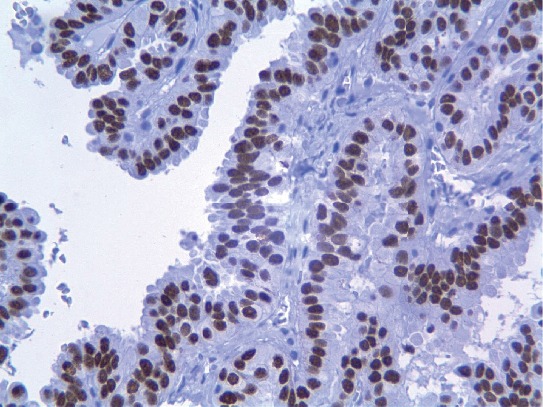
The nuclei of neoplastic cells were strongly and diffusely positive for TTF1.

**Figure 7:**
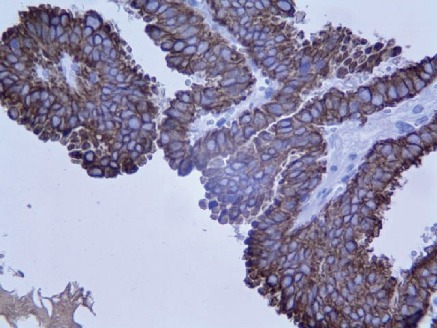
The cytoplasm of tumoral cells was strongly and diffusely positive for CK7 (×400).

A whole body scan revealed multiple highly metabolically active bony lesions in the chin, the left scapula, the anterolateral arc of the right 7th rib, the right iliac, and the right hip. In addition, both of the thyroid lobes and the isthmus were enlarged and nodular. A calcification was seen in the right nodule of the thyroid lobe, measuring 20 × 10 mm. A compressive effect of the right thyroid nodule on the trachea was obvious. Multiple adenopathies, with a maximum diameter of 14 mm, were evident in different parts of the neck's soft tissue, especially the right side. The tumoral cells were strongly and diffusely positive for TTF1 and CK7 (Figures 6 and 7). No immunoreactivity was seen for CK20.

**Figure 8:**
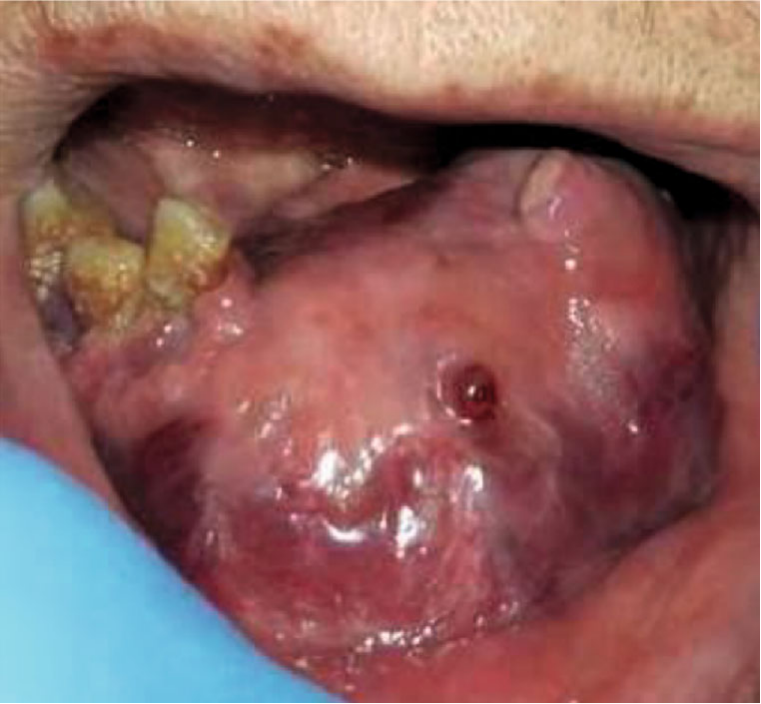
Large hemorrhagic mandibular mass 10 days following initial visit.

The microscopic features in combination with the IHC findings were consistent with “metastatic papillary thyroid carcinoma” (PTC). Within 10 days of the patient's first visit, the size of the lesion had nearly doubled, and eating had become difficult for her. In addition, she had been suffering from bleeding of the lesion (Figure 8) and numb-chin syndrome.

The patient was referred to an oncologist for further evaluation and management. According to the decision made by the professional team, a palliative en bloc resection of the lesion was performed. Unfortunately, the patient died one month after the diagnosis.

## 3. Discussion

Metastasis to the jaws is a rare event, and its early diagnosis is difficult [1]. Factors other than tumor frequency may be involved in oral metastasis, such as biological behavior of cancer, aggressiveness, and predilection for specific oral sites [2, 3]. Metastatic tumors are more commonly found in older adults [1]. The patient in our case was also in her 6th decade of life. Although it has been stated that jaw metastases have a tendency to happen in men, thyroid carcinoma metastasis affects women with a much higher proportion [1]. Metastases to the jaws have a predilection for the mandibular molar/premolar region [1, 4]. However, the anterior region of the mandible was involved in the current case.

Clinical signs and symptoms of jaw metastasis may include pain, swelling, tooth mobility, pathologic fracture, and paresthesia, but it may be asymptomatic [2]. The present case showed a painful swelling with teeth mobility and numb-chin syndrome. Mandibular metastasis with mental nerve involvement may cause numb-chin syndrome [6]. Irani [1] in her systematic review found that anesthesia and paraesthesia were seen in 90% of cases. Thus, it should be considered as a potential sign of metastasis to the mandible from an occult primary tumor [6]. Hirshberg et al. [4] mentioned that about 14% of patients with jaw metastasis had teeth extractions in the metastatic area, with an average time of 2 months between the extraction and the metastatic diagnosis. Our patient also lost her mandibular left central and lateral incisors before the disease was diagnosed. Oral metastasis usually shows simultaneous involvement of multiple body sites, especially the bones [2, 5]. Similarly, widespread skeletal metastasis was observed in this patient.

Most of the previously reported metastatic thyroid carcinomas of the oral cavity had been follicular thyroid carcinoma (FTC), and metastatic PTC was very rare [5, 7, 8]. Hurtle cell, poorly differentiated, and medullary carcinomas have been also reported in the mandible [5]. PTC is the most common microscopic variant of thyroid cancer. Moreover, it has a minimal potential for distant metastasis and tends to spread via lymphatic invasion [2, 5, 6]. It demonstrates an asymptomatic slow-growing thyroid mass, which is sometimes discovered incidentally [5]. Distant metastasis occurs in about 7-23% of patients with thyroid carcinoma [6]. Classic osseous regions for thyroid metastasis include the sternum, the vertebrae, and the pelvis [2, 5]. Metastatic PTCs or FTCs usually demonstrate an ill-defined radiolucent lesion in radiographs [5]. A PTC that shows intermediate clinical characteristics between PTC and FTC is considered a follicular variant of PTC (FV-PTC). In microscopy, it demonstrates a follicular pattern with the ground-glass nuclear features of PTC. Bone metastasis in the absence of lymphadenopathy in the setting of PTC may increase the suspicion of FV-PTC [2]. However, in the current case, both the incisional and the excisional biopsy showed a pure PTC pattern. The clinical signs of FTC or FV-PTC may be similar to arteriovenous malformations because they are highly vascular [2, 9]. Our patient also complained about bleeding from the lesion. Thyroid neoplasms are CK7+/CK20-. The panel of antibodies for thyroglobulin, TTF-1, CK7, and CK20 is valuable when the thyroid origin of a metastatic tumor is a possibility [10]. The current case also showed a CK7+/CK20- and TTF-1+ pattern. Distant metastasis has a crucial role in the patients' management, and oral metastasis is a therapeutic challenge for clinicians [10]. It ranges from palliation to several combinations of surgery, radioactive iodine, radiotherapy, and chemotherapy [2]. In our patient, palliative surgery was performed because of the huge mass and its bleeding. The 10-year survival rate for metastatic thyroid carcinomas is 13-21% [6]. Unfortunately, the present case died 1 month after the diagnosis.

Our literature review of metastatic thyroid carcinomas (PTC and FV-PTC variant) to the oral cavity (with sufficient information) resulted in 26 cases from 16 articles including our case. The demographic features are summarized and presented in Table 1 [2, 5, 7–22]. The mean age was 54.4 (ranging from 13 to 69 years). These malignancies occurred most often in the sixth and seventh decades of life, only 3 patients were under the age of 40, and no patient was found in the 8th decade of life. Nikitakis et al. [5] reported that the mean age of the metastatic thyroid carcinoma patients was 60.6 years with a tendency to occur in the 7th decade. Considering the age of occurrence for the primary thyroid carcinoma, which is about 40 to 50 years old, an increase in the mean age of metastasis is related to the amount of time that metastasis needs to develop and be detected [5]. There was a striking female predilection (88.46%) with a ratio of 7 : 66. This high tendency towards females is associated with the high incidence of thyroid carcinoma in women [5]. Among 25 patients with available tumor locations, the mandible was the most common site (80%). Other sites of involvement were the maxilla and the tongue. The tendency for spreading to the mandible may be associated with its greater volume of hematopoietic tissue that delivers easy access to neoplastic cells [18]. They tend to be more common in the jaws in comparison to oral soft tissues (4%). PTC and its FV-PTC variant comprised 53.84% and 46.15%, respectively. Among the 19 cases with available data about the time of metastasis, 42.10% were not aware of their primary cancer. Nikitakis et al. [5] in their review found that oral metastasis had been reported as the first sign of an unknown primary thyroid carcinoma in more than half of the metastatic thyroid carcinoma cases.

## 4. Conclusion

Although very rare, PTC can metastasize to the oral cavity. Oral metastasis may be the first symptom of a malignancy in other parts of the body. This emphasizes the role of oral professionals in identifying metastatic lesions. CK7/CK20 can be helpful in cases where the primary location of the metastatic tumor is unknown. Metastatic PTC/FV-PTC tends to occur in females, with a very high proportion, most often in the sixth and seventh decades of life. Additionally, there is a striking predilection for mandibular involvement. Reporting rare cases such as this one, together with a review of the pertinent literature will expand the body of knowledge about these uncommon lesions.

## Figures and Tables

**Table 1:** Published case reports of metastatic PTC and FV-PTC.

No.	References	Sex/age (years)	Location	Pathologic findings	Metastases found before primary tumor
1	Nishimura et al. [11]	F/51	Man	FV-PTC	Yes
2	Markitziu et al. [12]	F/69	Man and parotid	FV-PTC	Yes
3	Erdag et al. [13]	F/53	Man and infratemporal fossa	FV-PTC	No
4	Colella et al. [14]	F/50	Man	PTC	No
5	Liu et al. [15]	M/66	Man	FV-PTC	No
6	Tamiolakis et al. [16]	F/69	Man	PTC	N/A
7	Antunes and Antunes [7]	F/13	Max	PTC	N/A
8	Nikitakis et al. [5]	M/63	Max	PTC	No
9	Fatahzadeh et al. [2]	F/43	Max	FV-PTC	No
10	Seoane et al. [8]	F/58	Tongue	PTC	Yes
11	Seoane et al. [8]	F/69	Man	PTC	No
12	Muttagi et al. [17]	M/60	Man	FV-PTC	N/A
13	Muttagi et al. [17]	F/63	Man	FV-PTC	N/A
14	Muttagi et al. [17]	F/44	Man	PTC	N/A
15	Muttagi et al. [17]	F/35	Man	PTC	N/A
16	Muttagi et al. [17]	F/51	Man	FV-PTC	N/A
17	Kumar et al. [9]	f/58	Man	FV-PTC	Yes
18	Bhansali et al. [22]	F/60	Max	FV-PTC	Yes
19	Nawale et al. [18]	F/60	Man	PTC	No
20	Nawale et al. [18]	F/42	Oral	PTC	Yes
21	Nawale et al. [18]	F/50	Man	PTC	No
22	Nawale et al. [18]	F/56	Man	PTC	No
23	Anajar et al. [19]	F/52	Man	PTC	Yes
24	Ambelil et al. [20]	F/69	Man	FV-PTC^∗^	No
25	Bingol et al. [21]	F/33	Man	FV-PTC	No
26	Current case	F/56	Man	PTC	Yes

^∗^Man: mandible; Max: maxilla.
